# Visual Localizer: Outdoor Localization Based on ConvNet Descriptor and Global Optimization for Visually Impaired Pedestrians

**DOI:** 10.3390/s18082476

**Published:** 2018-07-31

**Authors:** Shufei Lin, Ruiqi Cheng, Kaiwei Wang, Kailun Yang

**Affiliations:** State Key Laboratory of Modern Optical Instrumentation, Zhejiang University, Hangzhou 310027, China; linshufei@zju.edu.cn (S.L.); rickycheng@zju.edu.cn (R.C.); elnino@zju.edu.cn (K.Y.)

**Keywords:** assisted navigation, place recognition, topological localization, impaired vision, convolutional neural networks, deep feature, network flow, data association graph

## Abstract

Localization systems play an important role in assisted navigation. Precise localization renders visually impaired people aware of ambient environments and prevents them from coming across potential hazards. The majority of visual localization algorithms, which are applied to autonomous vehicles, are not adaptable completely to the scenarios of assisted navigation. Those vehicle-based approaches are vulnerable to viewpoint, appearance and route changes (between database and query images) caused by wearable cameras of assistive devices. Facing these practical challenges, we propose Visual Localizer, which is composed of ConvNet descriptor and global optimization, to achieve robust visual localization for assisted navigation. The performance of five prevailing ConvNets are comprehensively compared, and GoogLeNet is found to feature the best performance on environmental invariance. By concatenating two compressed convolutional layers of GoogLeNet, we use only thousands of bytes to represent image efficiently. To further improve the robustness of image matching, we utilize the network flow model as a global optimization of image matching. The extensive experiments using images captured by visually impaired volunteers illustrate that the system performs well in the context of assisted navigation.

## 1. Introduction

In the world, 253 million people are estimated to be visually impaired, of whom 36 million people are totally blind [1]. The majority of visually impaired people, especially those in China, still use simple and conventional assistive tools, e.g., white canes. The extreme lack of assisted navigation approaches is not a rare situation among visually impaired people. According to our long-term observation and investigation, one of the most urgent demands for people with impaired vision lies in outdoor navigation with the goal to reach their destinations. Specifically, localization (i.e., locating oneself during an outdoor tour) is the critical session of outdoor navigation.

Thanks to the proliferation of intelligent devices and mobile Internet, the visually impaired people get access to coarse GNSS localization using mobile navigational applications on any ordinary smart phone. However, the outdoor localization error of consumer-level GNSS module is around 10–20 m, which is even worse under some severe weather conditions [2]. The errors of GNSS positioning is not so critical for sighted people, in that the visual capability helps to localize themselves and to reach the desirable place. However, things go differently for the visually impaired people. Imagining a common scenario that a person with visual impairments stands at the vicinity of a turning, it is tough for her or him to locate the exact position of turning relying merely on inaccurate GNSS localization. Therefore, the localization precision of those applications, which primarily localizes users relying on GNSS signals, is insufficient to meet the practical demands of visually impaired people. Obviously, applying a localization approach with less error to the practical navigational assistance is of vital importance for alleviating the potential hazards caused by inaccurate positioning.

Visual localization or place recognition, which utilizes the similarity of visual information to ameliorate localization precision, is a feasible solution to address the localization issues of assisted navigation, in view that visual appearance is a natural clue to a place or location. In general, the places to be located or recognized are recorded as a priori knowledge (also called database images). Visual localization refers to querying the best matching image from database according to the newly-captured image (also called query images). However, it is possible that the visual information of a location varies substantially between query and database from multiple environmental variations, which is a challenging issue for achieving robust visual localization [3].

**Figure 1:**
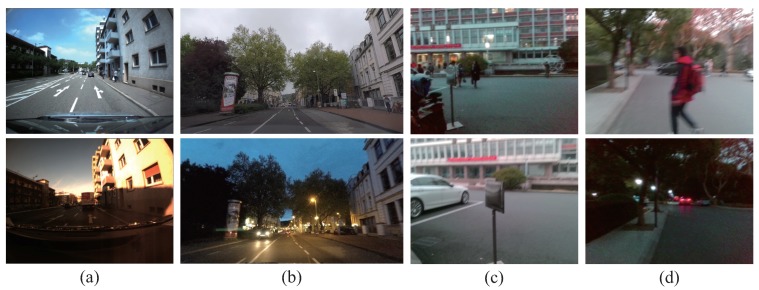
The exemplary query images (the upper row) and corresponding database images (the lower row) in: (**a**) Bonn dataset [8]; (**b**) Freiburg dataset [9]; and (**c**,**d**) the customized dataset.

The majority of visual localization approaches are designed for autonomous vehicles or robots [4,5,6,7]. In those scenarios, the typical environmental changes are shown in Figure 1a,b. Meanwhile, the issues of visual changes in localization for assisted navigation are shown in Figure 1c,d. The visual localization approaches applied to assisted navigation confront various environmental changes, some of which are similar to visual localization for autonomous vehicles. For example, the robustness against environmental changes, which are caused by changing illumination, diverse weather conditions and different seasons, should be the fundamental of localization reliability. However, other visual changes are exclusive in assisted localization, considering those changes are derived from cameras embedded in wearable devices. Thereby, the issues of visual changes are more complicated in assisted navigation.

To achieve precise visual localization in changing environments for people with visual impairments, we simultaneously face three critical challenges:

1. Viewpoint changes. As shown in Figure 1c, visually impaired people have no concept of viewpoint, and the wearable camera features large variations of lateral displacement and orientation during capturing visual images. Therefore, the database images and the query images of the same location most likely exhibit diverse viewpoints. In the context of autonomous vehicles, cameras are usually fixed on vehicles, thus the captured images share a relatively stationary viewpoint.
2. Appearance changes. As shown in Figure 1c,d, dynamic objects (e.g., vehicles, bicycles and pedestrians) in images result in appearance changes between database and query images. In the context of autonomous vehicles, the moving vehicles often maintain a secure distance from other vehicles or pedestrians, hence those dynamic objects are not salient in images. Moreover, the images captured by wearable cameras tend to be blurry due to shaking of carriers’ walking. If visual localization is deficient of the capability of recognizing the same location under different conditions, the practicability is largely limited.
3. Route changes. The visually impaired people may travel into a new place, which was not recorded in database. The localization system needs to not only reduce false alarms, but also re-localize the place when users return to the recorded route.

**Figure 2:**
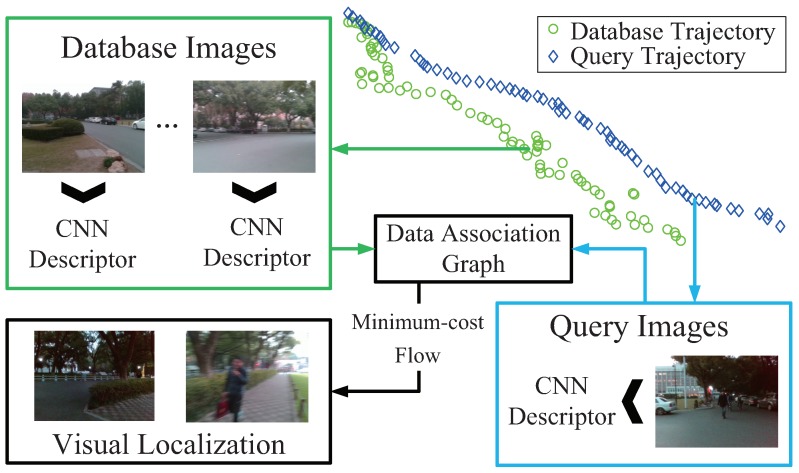
The general diagram of proposed Visual Localizer for visually impaired people. The entire framework is bipartite: ConvNet-based image description and network flow model-based image matching.

As presented in Figure 2, we propose Visual Localizer to solve those issues concerning accurate visual localization using merely color images, instead of using a priori pose information, such as GNSS signals or visual odometry. Visual Localizer is the core part of a visual localization-assistance system, which locates oneself by informing the user of the places visited before. In this paper, we only focus on the core part of an visual localization-assistance system, i.e., robust image matching between database images and query images.

Proposed for the context of visual assisted localization, Visual Localizer is composed of the customized ConvNet descriptor and the global optimization of image matching. To choose an outstanding ConvNet descriptor, a set of comprehensive comparisons is carried out between five state-of-the-art ConvNets on the performance of visual localization. With the optimal descriptor, the matching results of database and query images are organized by data association graph, and are subsequently optimized globally by network flow model (addressing a minimum-cost problem in the data association graph). The contributions of the paper lie in:

- Providing comprehensive evaluation of ConvNets on visual assisted localization. Aiming at practical scenarios of visual localization for the visually impaired, we perform layer-by-layer comparisons in five prevailing ConvNets on the problems of environmental changes, and analyze the description capability of layers in different levels.
- Proposing a visual localization framework—Visual Localizer. Considering the comprehensive comparison of different ConvNets, a lightweight ConvNet-based descriptor is put forward to depict holistic information of images. The possible image matching results are organized as a data association graph, based on which a minimum-cost flow problem is addressed to obtain optimal matching results and refuse potential mismatching images. In addition, the ConvNet descriptor and global optimization do not require training or tuning for specific environments.
- Adaptability to real-world scenarios. Visual Localizer is tested sufficiently both on public datasets and in practical environments, which demonstrates the robustness against viewpoint changes, appearance changes and route changes. The database and query images used in the real-world experiments are captured by visually impaired volunteers. Beyond the domain of assisted navigation, the system can also be applied to autonomous driving and robotics context.

We discuss related work in Section 2, and describe the system configuration of Visual Localizer in Section 3. In Section 4, the evaluation results of ConvNet-based image descriptors and localization performance are elaborated. Finally, we conclude the paper along with an outlook to future work in Section 5.

## 2. Related Work

In this section, we discuss related work on localization approaches applied to assisted navigation as well as visual place recognition.

### 2.1. State-of-the-Art Assisted Localization

In the research field of assisted navigation, localization systems aim to locate the user by exploiting different types of sensors. Beacon-based localization approaches locate the user by the proximity to a beacon, which could be Bluetooth beacons [10], RFID beacons [11], etc. Beacon-based localization is usually applied to indoor environments, and is not very suitable for outdoor localization. Although we could deploy RFID beacons in outdoor environments, it still has some drawbacks. The deployment of passive RFID tags for each person with visual impairments even on his or her familiar routes is still a great deal of work, if the outdoor localization systems are broadly used. Ivanov [12] proposed a simulated indoor navigation system, which requires a priori knowledge of building topology and geometry. Al-Khalifa and Al-Razgan [13] constructed building map based on floor plans and located the user at key positions represented by QR code. With a similar purpose, Li et al. [14] aligned visual features derived from camera images with semantic maps derived from architectural floor plans to achieve localization. Murata et al. [15] proposed a smart phone-based indoor localization system for blind navigation to address the localization issues in large-scale environments. Apparently, those localization approaches relying on architectural floor plans are not flexible enough, thus their applications are usually constrained, e.g., within or around public architectures.

The striking development of visual sensors and computer vision spurred the research community of assisted navigation to orient to visual localization. To perceive the locations once visited, compressive sensing [16] and GIST features [17] are applied to scene recognition. Nevertheless, those approaches are not designed to locate users, hence the localization performance (i.e., the localization precision and robustness against environmental changes) is not guaranteed. Fusco et al. [18] proposed a self-localization approach based on a street-view panorama and an aerial image. The algorithm locates visually impaired people by extracting crosswalk stripes from both images, so it is only valid at street intersections. Brilhault et al. [2] achieved precise localization by combining landmarks detected from visual images with corresponding labels in GIS. Unfortunately, localization is constrained around the limited landmarks, which need to be labeled artificially in GIS.

### 2.2. Visual Localization System

The concept of visual localization was coined originally by the community of autonomous robots and vehicles, which has achieved plenty of visual localization systems. As a prevalent mapping and localization approach, SLAM [19] is widely used in the research area of robotics to build the metric map and localize the current position. For instance, ORBSLAM2 [20] leveraged visual odometry to track unmapped regions and matches with map points that allow for zero-drift localization. The visual information is converted into image key points stored in dense or sparse metric maps. SLAM-based localization approaches, which are complicated to achieve in dynamic environments, pay more attention to mapping and localization in ambient environments, hence it is not applicable to facilitate localization in a long-range outdoor navigation.

Visual localization based on topological maps [3], which remove metric information from metric maps but maintain transition information, is usually adopted to achieve unconstrained outdoor localization. In those system, both database images (a priori knowledge) and query images are processed by image representation, and database images are organized in a topological structure. Those localization approaches perform well on the precise localization of autonomous robots and vehicles, but their adaptability to assisted navigation for visually impaired people still needs to be validated.

In the next section, we review the prevailing visual localization approaches in terms of image representation and image matching.

#### 2.2.1. Image Representation

Originated from scene categorization or object classification, various image descriptors have been taken advantage of in visual localization. Bag-of-word place recognition module is applied to achieve loop detection in ORBSLAM2 [20]. Due to the large dimensions of BoW descriptor, inverse index of vocabulary tree is used to quickly access the nearest neighbors of query images [21], and the distance between any two descriptors are not obtained directly, which hampers the building of topological structure. With much smaller dimensions, the global descriptor LDB is used by Arroyo et al. to implement a visual localization algorithm [22]. Similarly, HOG descriptors that are extracted from image cells served as the holistic representation of scenes [23]. However, those descriptors are sensitive to the changing pose and FOV of cameras, which is difficult to keep stable on wearable devices. Moreover, local features, such as SURF [24], are also used to match images and achieve localization, but the performance of localization degrades with the high computational complexity and the low recall rate.

Features learned from deep neural networks have recently been used as robust feature detectors for visual localization. Motivated by their ability to learn generic features that are transferable to a variety of related but different visual tasks, some studies utilized ConvNet features as holistic image descriptors. Recent studies on deep ConvNets have concentrated on improving the classification accuracy. AlexNet [25], VGG [26], GoogLeNet [27] and ResNet [28] achieve better performance as the size and depth become larger. However, all of the networks above have large scale of parameters, which pose a huge challenge for deploying these deep learning algorithms on the mobile devices and embedded applications with limited computational resources. The compression and acceleration of ConvNet models have become one of the most important research fields in both academia and industry. For instance, SqueezeNet [29], MobileNet [30] and ShuffleNet [31] achieve AlexNet-level accuracy with fewer parameters. There are several major gaps in our knowledge and capability regarding deep learning and place recognition. It is still unclear what kind of ConvNet specifically for the task of place recognition will yield the best robustness. Currently, few studies have investigated and analyzed the performance of different ConvNets for visual localization, including standard ConvNets and lightweight ConvNets mentioned above. It is worth mentioning that the mid-level features are relatively more robust against the appearance changes, while the higher-level features exhibit robustness against viewpoint changes [32,33,34].

By achieving impressive localization accuracy in spite of significant environmental changes, ConvNet landmark-based approach [34] has attracted the attention of several research communities including autonomous vehicles and robotics. It takes advantage of Edgebox [35], a landmark proposal system, and AlexNet to extract features from the landmark proposals. Hou et al. [36] leveraged MRoI pooling to exploit multi-level and multi-resolution information from multiple convolutional layers, and then fused them to improve the discriminative capacity of the final ConvNet features.

Other studires rely heavily on the outstanding discrimination power of ConvNet features between images. PoseNet [37] presented a framework of computing continuous pose directly from appearance, and replaced all three softmax classifiers of GoogLeNet with affine regressors. NetVLAD [38] was designed for place recognition that aggregates mid-level convolutional features extracted from the entire image into a compact single vector representation amenable to efficient indexing. However, none of the above networks have been analyzed comparatively regarding the adaptation to visual changes in the application of assisted navigation.

#### 2.2.2. Image Matching

The pure image retrieval is a straightforward approach to match query images with database images, which ignores the transition information between adjacent database images and regards them as equal during image retrieval. Dzulfahmi and Ohta [39] evaluated the image retrieval performance based on conventional local features. In view that topological structure is lacking in the algorithm, the parameters of the algorithm need to be refined carefully for precise localization. Besides, to cope with multiple changes between query images and database images, robust image representation is far from sufficient, and the topological structure among the images is required to achieve precise localization.

Instead of querying the most likely location from a single image, SeqSLAM [40] achieves localization by recognizing coherent sequences of best matching images. In a similar way, ABLE [7] concatenates adjacent image descriptors to build a final descriptor and located the current position by matching sequential images matching. Identifying localizations as sequences of images is not effective all the time, considering the query images are possibly recorded at different speed with those in database images.

The two kinds of solutions above merely make use of several image matching results to optimize localization performance, and are not robust against fallible image matching. To address the issues, the global optimization of images matching results is necessary. As a feasible topological structure to globally optimize the matching results between query images and database images, data association graph [23] outperformed SeqSLAM [40] in higher localization precision.

Notably, to the best of our knowledge, no visual localization solution aims or manages to promote the localization precision of visually impaired people under unconstrained outdoor environments. Based on these observations, we aimed to design a visual localization framework for assisted navigation that can locate the visually impaired user in real-world scenarios.

## 3. Visual Localizer

**Figure 3:**
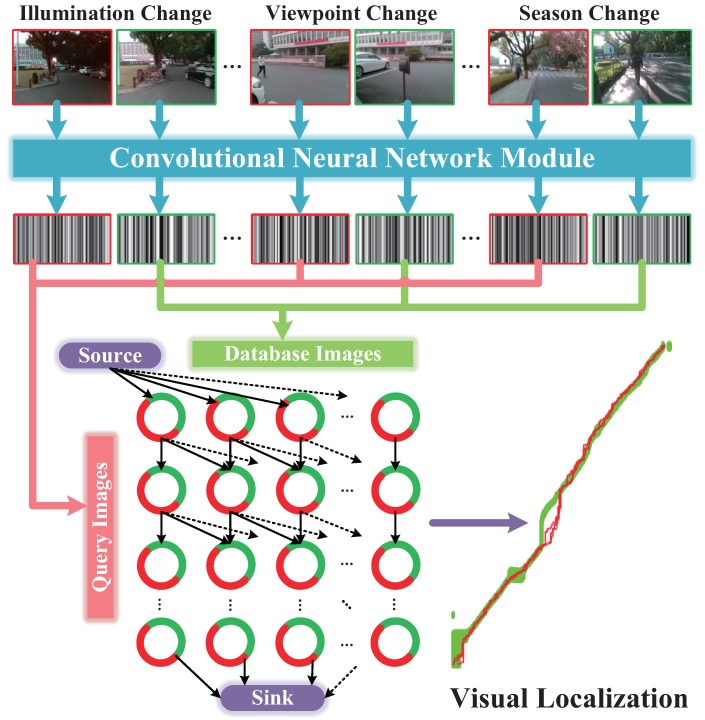
The flow chart of the proposed Visual Localizer.

In this section, we elaborate the proposed Visual Localizer, a visual localization framework which is robust against viewpoint, appearance and route changes. The detailed flow chart of Visual Localizer is shown as Figure 3. The entire visual localization system involves two successive sessions: image representation and matching optimization. In the former session, database images (labeled with red in Figure 3) and query images (labeled with green in Figure 3) featuring different visual changes are depicted by the optimal ConvNet layers. After that, the raw descriptors are processed by selection compression to reduce the dimension of descriptors. In the latter session, the database descriptors and query descriptors are utilized to organize a directed data association graph, where a node denotes a pair of matching combination and an edge weight directed to a node denotes the similarity of that pair. The optimized localization results are obtained by addressing a minimum-cost flow problem of the data association graph.

### 3.1. ConvNet-Based Image Representation

Database images and query images feature different visual changes, including viewpoint changes, illumination changes, season changes, route changes, etc. In this part, we select the optimal ConvNet descriptor to robustly depict images captured at the same location but with various changes. To achieve efficient image matching, the optimal descriptors are compressed to a smaller scale.

Neuron weights over all stages of ConvNets are trained on datasets featuring artificially labeled images, such as ImageNet dataset [41] or Places dataset [42]. After that, the ConvNet has acquired the capability of distinguishing objects of different classes. Pre-trained convolutional layer has a set of learnable filters that are of equal depth to the input volume. By sliding each filter over the input volume and performing the convolution operation at every position, we obtain a two-dimensional array called feature map, which contains simple structural features or semantic features extracted by the corresponding filter. For each filter, we get a distinct feature map, which means that the number of feature maps at a convolutional layer is determined by the number of filters of that layer. Finally, the output volume is formed by concatenating all feature maps along the depth dimension. However, some filters failed to be learned to deliver any feature representation and the feature maps are totally meaningless. By vectorizing the feature maps to one-dimensional feature vectors and concatenating the vectorized features obtained by different layers, we generate a holistic image descriptor, which aims to be robust to environmental changes of the whole input image instead of a part of input image. The appearance changes fall into different aspects: viewpoint, illumination, season, etc.

To select a optimal image descriptor robustly against environmental changes, a high- performance ConvNet model is selected from off-the-shelf ConvNet models (i.e., AlexNet, VGG16, GoogLeNet, SqueezeNet and MobileNet) in the consideration of robustness and computational cost. In the following, we introduce the five ConvNets with the corresponding architecture, which are various in filter size, convolution operation, width and depth of the network, etc.

#### 3.1.1. AlexNet

**Figure 4:**
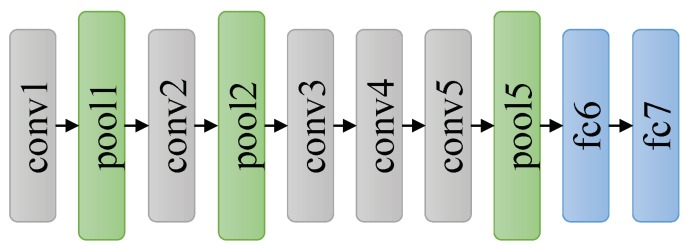
The architecture of AlexNet.

Benefitting from large datasets and parallel computing technology, AlexNet first achieved its success on object classification task in 2012 [25], which really changed the field of deep learning in the computer vision community. As shown in Figure 4, AlexNet consists of five convolutional layers followed by two fully connected layers. The sizes of convolution filters at the first and the second convolutional layer are 11 × 11 and 5 × 5, respectively, but the size of convolution filters at the rest layers is 3 × 3.

#### 3.1.2. VGGNets

**Figure 5:**
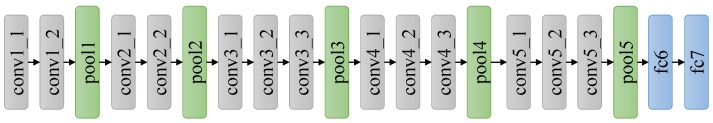
The architecture of VGG16.

To further promote the classification accuracy, VGGNets increase the depth of the networks by simply stacking standard convolutional layers. VGGNets replace large filters with 3 × 3 filters, which are the smallest size to capture the information of left, right, up, down and center portion of input layer. As shown in Figure 5, a stack of 13 convolutional layers are followed by two fully-connected layers. Using multiple convolutional layers with smaller filter size not only reduces the quantity of parameters, but also performs more nonlinear mappings thus increases the fitting ability of the network.

#### 3.1.3. GoogLeNet

**Figure 6:**
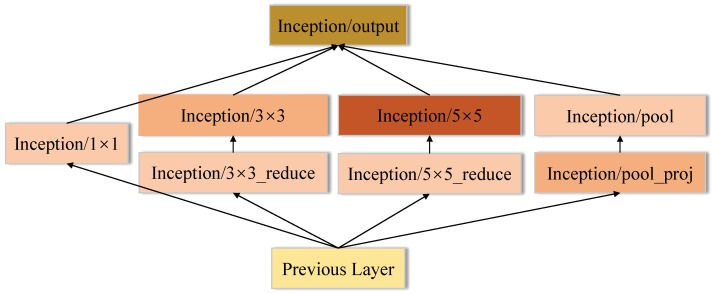
The architecture of Inception module.

**Figure 7:**
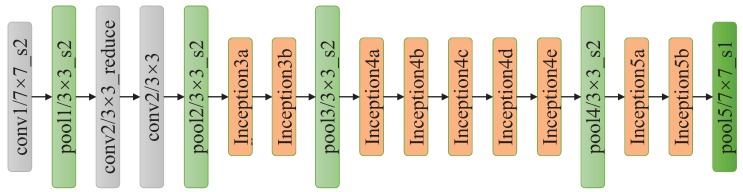
The architecture of GoogLeNet.

Another way to get higher classification accuracy is to widen the networks, hence GoogLeNet constructs an Inception module, which includes four parallel operations: 1 × 1 convolution, 3 × 3 convolution, 5 × 5 convolution and max pooling. Then, the output of these four operations are concatenated along the depth dimension and fed to next layer. GoogLeNet effectively increases the network width, but causes a boost in space and time complexity due to the large computational cost of 5 × 5 convolution operation. To reduce the side-effect of large convolution filters, the solution is to add 1 × 1 convolution to reduce the dimension of input channels followed by the standard convolution as shown in Figure 6. As presented in Figure 7, GoogLeNet includes three convolutional layers and nine Inception modules. Even though GoogLeNet is deeper and wider than VGGNets, the computational cost and memory consumption of GoogLeNet is much smaller than VGGNets.

#### 3.1.4. SqueezeNet

**Figure 8:**
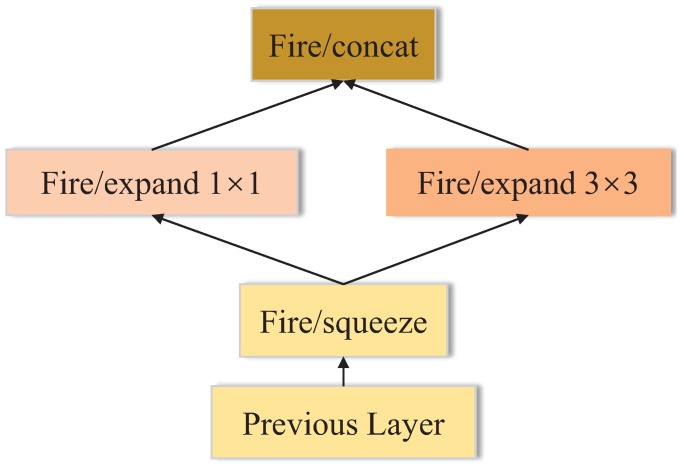
The architecture of Fire.

**Figure 9:**
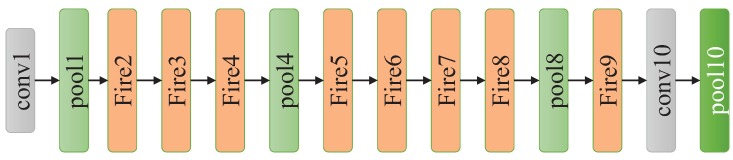
The architecture of SqueezeNet.

To reduce the number of parameters, SqueezeNet proposes the Fire module comprising a squeeze convolutional layer, which has only 1 × 1 filters since a 1 × 1 filter has much fewer parameters than a 3 × 3 filter. Then, the layer is fed into an expand layer that has a mix of 1 × 1 and 3 × 3 convolution filters as shown in Figure 8. The squeeze layer is responsible for compressing the input channels while the expand layer utilizes different filters to extract features, which is similar to the Inception module of GoogleNet. As presented in Figure 9, SqueezeNet begins with a standalone convolutional layer, followed by eight Fire modules and three max pooling layers, and ends with a final convolutional layer. SqueezeNet has only 1.25 million parameters, while maintaining competitive accuracy.

#### 3.1.5. MobileNet

**Figure 10:**
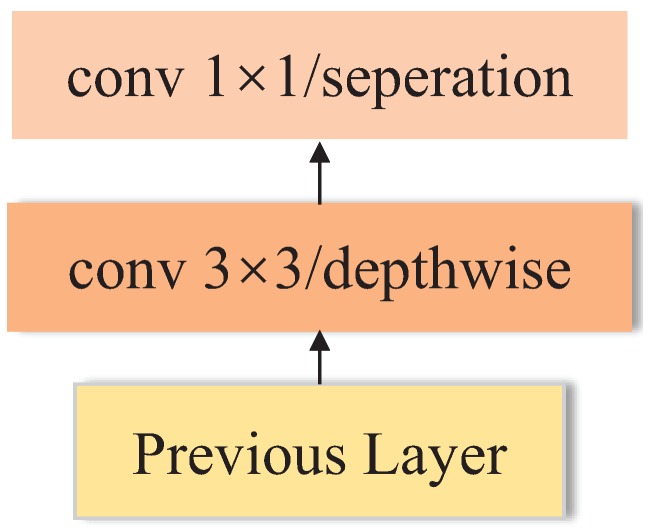
The architecture of depthwise separable convolution.

**Figure 11:**
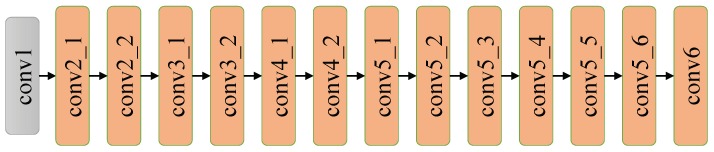
The architecture of MobileNet.

Assuming that cross-channel correlations and spatial correlations can be mapped completely separately, MobileNet utilizes depthwise separable convolutions instead of standard convolutions to extract features, thus it reduces the computational complexity in a way that is different from SqueezeNet. As presented in Figure 10, depthwise convolution splits convolution into two separate layers: depthwise convolutional layer and pointwise convolutional layer. Depthwise convolution performs lightweight filtering by applying a single convolutional filter per input channel. Pointwise convolution is responsible for building new features through computing the combinations of depthwise convolution outputs. As shown in Figure 11, the network contains a standalone convolutional layer followed by 13 depthwise separable convolutional blocks without pooling layers. Downsampling is engineered into the architectures by setting the stride in some of the convolutional layers. Recently proposed MobileNet v2 [43] is based on an inverted residual structure and has better performance on recognition task than MobileNet v1.

### 3.2. Global Optimization of Image Matching

Network flow model based on data association graph was used to achieve robust visual localization across seasons [23]. In this paper, data association graph is also applied to the global optimization of Visual Localizer.

**Figure 12:**
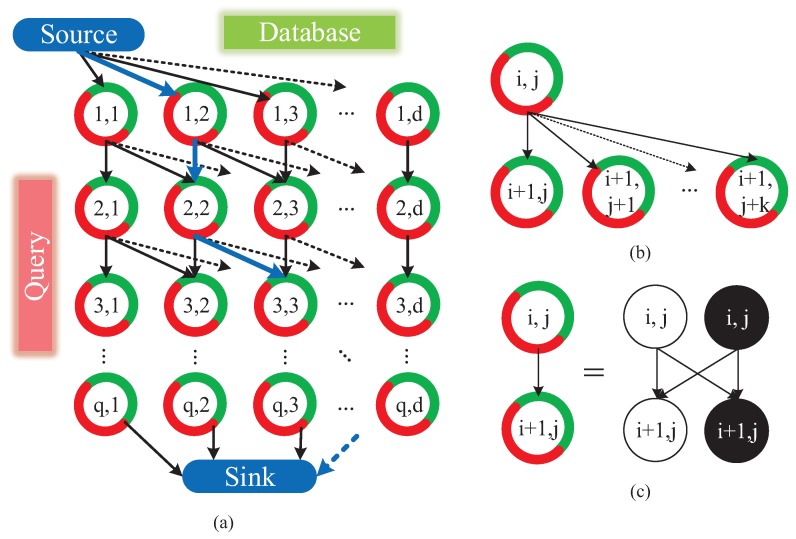
The global optimization model of Visual Localizer: (**a**) data association graph is comprised of “nodes” and directed arcs which are derived from database and query descriptors; (**b**) the arcs connect a “node” (i,j) with k+1 adjacent “nodes” (i+1,j+s), where 0≤s≤k and j+s≤d; and (**c**) a “node” in graph involves a matching node (white node) and a hidden node (black node), while the arc interconnecting “nodes” is composed of edges interconnecting with matching and hidden nodes.

Herein, we interpret the configuration of data association graph briefly. As shown in Figure 12a, the data association graph is a directed graph, which consists of three elements:

- Node. Nodes fall into three types: source node, sink node and ordinary “node”. The ordinary “node” (i,j) reflects the state that the *i*-th query image matches with *j*-th database image. As Figure 12c showing, each ordinary “node” is actually comprised of a matching node and a hidden node, which denotes the matching or mismatching state of two images, respectively.
- Edge. We define a directed connection between two “nodes” as an arc, which is represented with an arrow in Figure 12a,b. Meanwhile, as shown in Figure 12c, a directed connection between actual nodes is defined as an edge. The arc is the encapsulation of multiple edges. Source node connects with all of the nodes in the first row of graph. Similarly, sink node connects with all of the nodes in the last row of graph. As Figure 12b,c shows, the number of arcs originated from one ordinary node is equal to or less than k+1, and those arcs merely point to nodes in the next row of graph.
- Cost. Cost *w* is associated to each edge. The cost of edges associating with source or sink node is set to 0. The cosine distance [23] is utilized to measure the similarity of two images in a matching node. The cost of edges pointing to a matching node is the reciprocal of corresponding cosine distance. Moreover, the cost of edges pointing to a hidden node is set to *c*.

Minimum-cost flow problem [44] is to find a flow with minimal cost such that all the fluid flows from the source nodes to the sink nodes. In our cases, the only source node produces flow, meanwhile the only sink node consumes flow. The quantity of flow generated by the source node is set to *f*. The capacity of an edge, which denotes the number of units that can flow over that edge, is set to f+1 at edges interconnecting the hidden nodes and 1 at other edges. In our cases, the minimum-cost flow problem is solved by Google Optimization Tools [45]. The solutions to the problem yield a path in the graph, as blue arrows shown in Figure 12a. It is the nodes on the path that serve as optimized image matching result of visual localization.

Solving a minimum-cost flow problem of the proposed data association graph is arguably an effective way to achieve global optimization of single image matching. Compared with single image matching, network flow model fulfills image matching in the global level, hence it is robust to cope with more complicated localization scenarios. Due to hidden nodes, the localization algorithm can refuse bad image matching results.

## 4. System Evaluations and Experiments

In this section, we elaborate the experiments on promoting the performance of Visual Localizer. Firstly, robust ConvNet-based image descriptor is determined after evaluating different layers from five ConvNets. Subsequently, the parameters in the net flow model of data association graph are tuned for the optimal performance of image matching.

### 4.1. Datasets

Challenging datasets featuring various changes between query and database images are utilized to evaluate the performance of each layers derived from different ConvNets. The datasets that are used in this paper are listed below.

1. Gardens Point Walking dataset [32]. The Gardens Point dataset consists of three traverses of the Gardens Point Campus of QUT in Brisbane. Two subsets were captured. One was captured during the day, which forms viewpoint change (left vs. right). The other one subset was captured during the night, which forms illumination change (day vs. night).
2. Nordland dataset [46]. The Nordland dataset consisting of 10-h video footages of sceneries captured on the train in different seasons exhibits no viewpoint variations, and therefore allows testing the ConvNets on pure condition changes and appearance (across-season) changes. In our experiments, 400 images are extracted from the summer videos (as database images) and winter videos (as query images), respectively.
3. Bonn dataset [8]. Recorded by the car-mounted camera in Bonn city during different time, the Bonn dataset consists of 488 images (as database dataset) and 544 images (as query dataset). The query as well as database trajectory contains several revisits of the same places. The dataset features illumination changes and viewpoint changes.
4. Freiburg dataset [9]. Recorded by the car-mounted camera during different seasons, the Freiburg dataset consists of 361 images (as database dataset) and 676 images (as query dataset). The dataset captures significant perceptual and structural changes over the span of three years, which includes viewpoint changes and extreme seasonal variations. The database images and query images have the same start and end points. It is worth noting that the query images include the situation that the vehicle encounters red traffic light and stays for a while on the road.

### 4.2. Evaluation Criteria

Herein, we define true positives, false positives and false negatives, according to the consistency between ground truths and localization predictions.

- True positive (TP). The localization system matches the query image with a database image and the matching result is consistent with the ground truth.
- False positive (FP). The localization system matches the query image with a wrong image, which is different from the ground truth.
- False negative (FN). The localization system gives no response for a query image, but there are database images associated with the query image.

The performance of Visual Localizer is evaluated and analyzed in terms of precision and recall. As defined in Equation (1), precision is the proportion of true positives out of all predicted positives. Moreover, recall is the proportion of true positives to all of actual positives, as defined in Equation (2).

(1) $$Precision=\frac{TP}{TP+FP}$$

(2) $$Recall=\frac{TP}{TP+FN}$$

Considering both the precision and the recall, $F_{1}$ score is the harmonic average of the precision and recall. $F_{1}$ score reaches its best value at 1 and worst at 0.

(3) $$F_{1}=2\times \frac{Precision\times Recall}{Precision+Recall}$$

We inspect each individual layer of different ConvNets on viewpoint, illumination and cross-season performance. Each individual layer extracted from networks is taken as a holistic descriptor of input image, which is resized to 224 × 224 in advance. Originally in a float format, the descriptors extracted from ConvNets should be cast into a normalized 8-bit integer format.

(4) $$d_{int}=\left[d_{float}-min\left(d_{float}\right)\right]\frac{255}{max\left(d_{float}\right)-min\left(d_{float}\right)}$$

The length of the vectorized descriptors can be calculated as exposed in Equation (5), where $h_{i}$, $w_{i}$, and $d_{i}$ are the height, width and dimensions of each layer, respectively. *n* is the number of layers to be concatenated together.

(5) $$l_{feature}=\sum_{i=1}^{n}\;h_{i}\times w_{i}\times d_{i}$$

During the evaluation of ConvNet layers, the simplest single-image nearest-neighbor matching based on Hamming distance [47] is adopted as image matching strategy, so as to avoid the influence of image matching on the performance evaluation of ConvNets. All of the layers in different ConvNets are checked, and their performance are presented as precision–recall curves. The variable creating the precision–recall curves is the threshold of ratio test [32], which is the ratio of the distance of the best over the second best match found in the nearest neighbor search. The localization result of a query image is regarded as a true positive only if the image matching result passes the ratio test.

### 4.3. Performance Analysis and Comparison between Different ConvNet Layers

**Figure 13:**
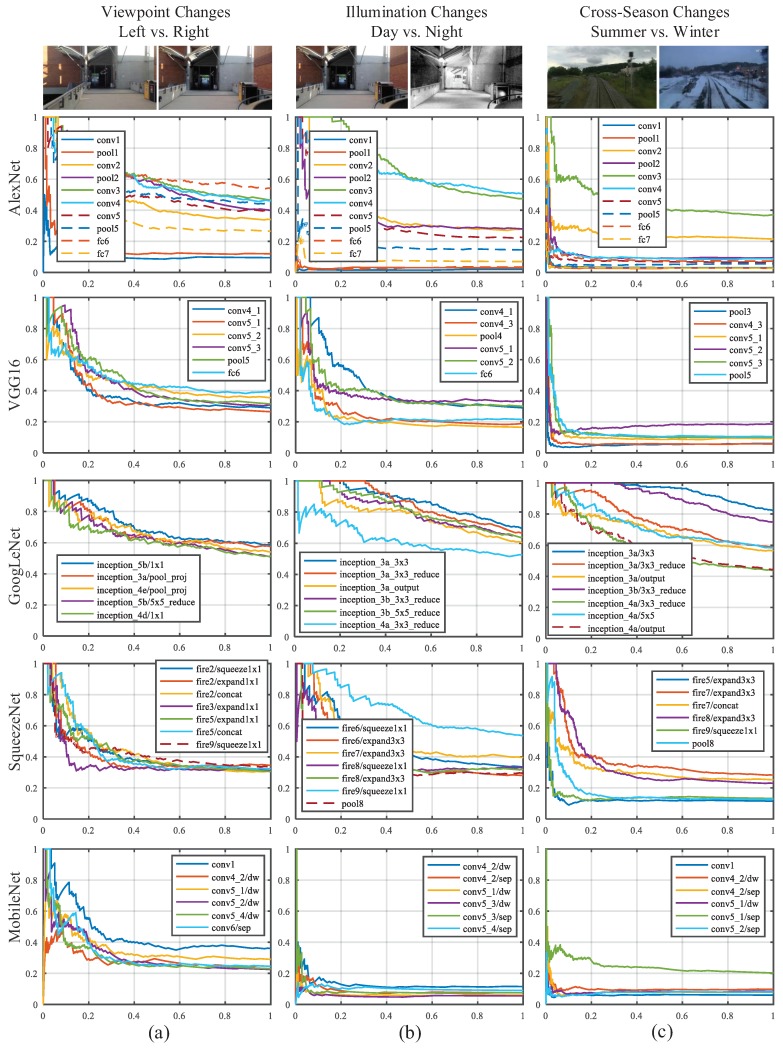
Precision–Recall curves comparing results about different layers of five ConvNets on different datasets: (**a**) comparison results about left vs. right in Gardens Point dataset; (**b**) comparison results about day vs. night in Gardens Point dataset; and (**c**) comparison results about summer vs. winter in Nordland dataset.

In this section, the detailed layer-by-layer analysis concerning visual localization performance is presented. To evaluate the performance of the prevailing ConvNets, we used the representative datasets featuring three aspects: viewpoint changes, illumination changes and cross-season changes. The precision–recall curves of different ConvNet layers on different datasets are shown in Figure 13.

AlexNet. As presented in Figure 13, the features extracted from the AlexNet have a similar behavior to the observation of [32]. The mid-level features derived from conv3 are most robust against appearance changes. Furthermore, conv3 achieves a precision of around 50% at 100% recall rate on the viewpoint change dataset, which is merely inferior to the performance of high-level fc6 of AlexNet.

VGG16. Features extracted from layers ranging from conv4 to fc6 of VGG16 have similar robustness against several changes, as the Top 6 results presented in Figure 13. However, the features have sub-optimal performance on viewpoint invariance and illumination invariance, in view that the precision at 100% recall rate are less than 40%. The poor performance on cross-season invariance illustrates that the features from single layer are not able to overcome all kinds of appearance variances. For example, the features extracted from conv4_1 display opposite performance between illumination invariance and cross-season invariance.

GoogLeNet. The Inception module was proposed to eliminate the influence of different size filters on the recognition task [27]. The extraordinary performance of GoogLeNet illustrates that using different filters simultaneously is able to allow the ConvNet to choose the most appropriate features for visual localization, as shown in Figure 13. On the viewpoint change dataset, the precision of Inception5b/1 × 1 at 100% recall rate is about 60%. The features from Inception3a/3 × 3 are most robust against both illumination and cross-season changes, and the precisions on illumination and cross-season changes dataset are more than 70% and 80% respectively. The second best feature for illumination invariance and cross-season invariance are Inception3a/3 × 3_reduce and Inception3b/3 × 3_reduce, respectively. We find that the filter size of most top-rank layers is 1 × 1. In other words, the capability of feature extraction is not proportional to the filter size. Instead, the feature maps of convolutional layers with small filter size (especially 1 × 1) have better performance on appearance invariance and viewpoint invariance.

SqueezeNet. The high-level features ranging from Fire6 to Fire9 are robust against appearance changes and mid-level features ranging from Fire2 to Fire5 are robust against viewpoint changes. Particularly, Fire9/squeeze1 × 1, consisting of 13 × 13 × 64 feature maps, performs well both on illumination and viewpoint invariance. However, it displays a limited performance on cross-season invariance. The precision at 100% recall rate of all the layers (except Fire9/squeeze1 × 1) are less than 40% on the three datasets because of the drastic compressing operation of squeeze layer, which might loses key attributes for visual localization.

MobileNet. The lightweight depthwise convolution of MobileNet not only requires less computational resources than fully convolution, but also retains higher accuracy on image recognition tasks compared with AlexNet and GoogLeNet [30]. Nevertheless, on the visual localization task, each layer of both MobileNet performs inferiorly compared with AlexNet and GoogLeNet. The depthwise separable convolutional block impedes information flow between different channels, which might result in the degradation of an individual convolution filter and weaken the representation of the corresponding feature map. It is the most critical reason the ability of feature extraction of the depthwise separable convolutional block is far worse than standard convolutional layer.

**Figure 14:**
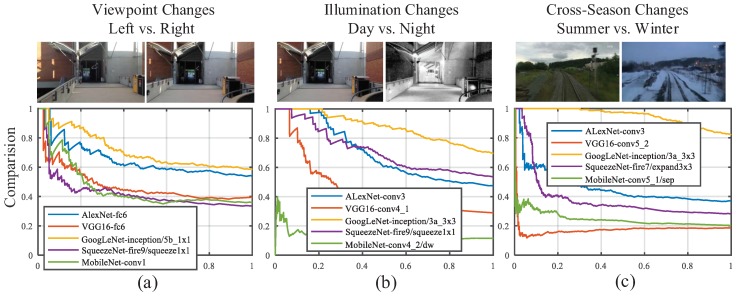
Precision–Recall curves comparing between the best result selected from each ConvNets on different datasets: (**a**) comparison results about left vs. right in Gardens Point dataset; (**b**) comparison results about day vs. night in Gardens Point dataset; and (**c**) comparison results about summer vs. winter in Nordland dataset.

Furthermore, the performance comparison between the best results selected from each ConvNet is also shown in Figure 14. In Table 1, we conclude the performance of each ConvNet trained on ImageNet datasets and the assessment on the three aspects. From the results of experiments, we summarize four conclusions:

1. In AlexNet, VGG16 and GoogLeNet, the features extracted from the mid-level layers are more robust against appearance changes, which is consistent with the conclusion made by Sünderhauf et.al. [32]. If the feature is illumination-invariant, it also exhibits season-invariant robustness, such as conv3 of AlexNet, conv4 and conv5 of VGG16 and Inception3a module of GoogLeNet. However, lightweight CovNets, such as SqueezeNet, seem contrary to the conclusions mentioned above.
2. As shown in Table 1, the object recognition accuracy on the ImageNet dataset of VGG16 and MobileNet are 71.5% and 70.6%, respectively, which are better than other ConvNets. However, the features from VGG16 and MobileNet have inferior performance on appearance invariance. It illustrates the fact that the performance on the object recognition is not completely transferable to the task of visual place localization.
3. As presented in Figure 14, the most layers of each ConvNet exhibit satisfactory precision on the viewpoint changes dataset, which illustrates the fact that convolutional layer features the nature of translation invariance. Given this insight, appearance changes will be paid more attention to in our selection of robust convolutional layer.
4. GoogLeNet has overwhelming advantages against other ConvNets because of best performance on both appearance invariance and viewpoint invariance as well as modest computational complexity. Based on this observation, we choose GoogLeNet as the optimal ConvNet, from which we select robust layers to depict images.

### 4.4. Visualization Analysis of Features Extracted from Different Levels of GoogLeNet

**Figure 15:**
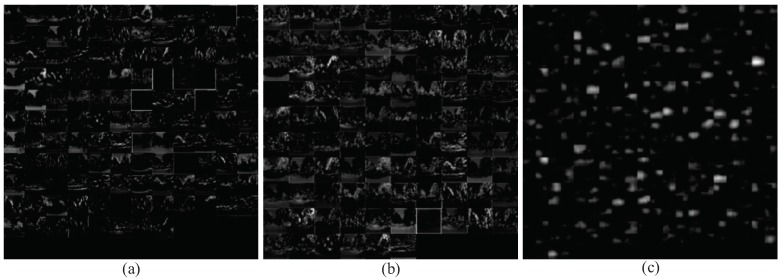
The feature maps extracted from different layers of GoogLeNet: (**a**) Inception3a/3 × 3 is comprised of 28 × 28 × 128 feature maps; (**b**) Inception3a/3 × 3_reduce is comprised of 28 × 28 × 96 feature maps; and (**c**) Inception5b/1 × 1 is comprised of 7 × 7 × 384 feature maps.

Low-level and mid-level feature maps have larger size than high-level feature maps. Lower-level features assemble simple but discriminating shape features such as oriented edges and colors, which benefits place recognition under severe appearance changes. Derived from the Freiburg dataset, feature maps of Inception3a/3 × 3 layer and Inception3a/3 × 3_reduce layer (the second best layer on illumination change) of GoogLeNet are shown in Figure 15a,b, respectively. Some feature maps focus on the structural parts which preserve the shape features of landmarks and trees in the environment, while some feature maps focus on the non-salient parts like the sky region in the image. The other feature maps have no response due to the corresponding filters failed to be learned to deliver any feature representation.

The high-level features are more abstract and semantically meaningful, but lose their ability to discriminate between individual places within the same semantic type of scenes. Figure 15c shows the feature maps of Inception5b/1 × 1 layer in GoogLeNet, which are derived from the same image of the Freiburg dataset. It is shown that the feature maps lose the details of the environment but still have the ability to retain important semantic information to a certain extent. Meanwhile, the position of semantic pixels always changes with the variations of camera pose, which refers to lateral displacement, orientation and scale.

### 4.5. Concatenation and Compression

**Figure 16:**
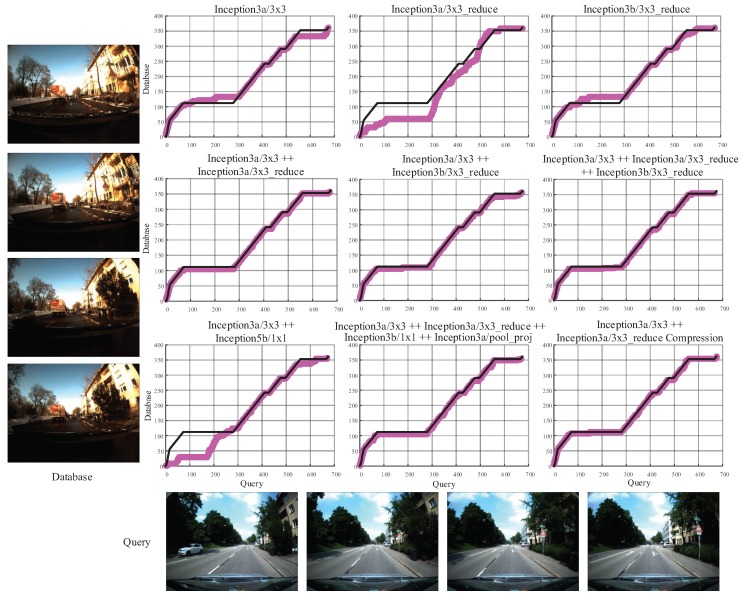
The performance of different layers and different combinations of layers in GoogLeNet on the Freiburg dataset. In detail, the Inception3a/3 × 3 (28 × 28 × 128) is most robust against illumination changes and cross-season changes. The Inception3a/3 × 3_reduce (28 × 28 × 96) and Inception3b/3 × 3_reduce (28 × 28 × 128) are second best layer for illumination invariance and cross-season invariance, respectively. The Inception5b/1 × 1 (7 × 7 × 384) and Inception3a/pool_proj (28 × 28 × 32) are best and second best layer for viewpoint invariance, respectively. The black line denotes the ground truth matches, and the mulberry line denotes the matching results predicted by Visual Localizer.

Because the features from single layer are not able to overcome all kinds of changes, it is obvious that utilizing concatenated layers rather than a single layer as the holistic image descriptor gets more robust performance. However, it is necessary to select and concatenate a handful of layers that perform robust against changes, as the computational cost of image matching is proportional to the dimension of holistic image descriptor. As shown in Figure 16, we compare the performance of different layers of GoogLeNet and different combinations of layers on the Freiburg dataset. The combination of the Inception3a/3 × 3 and Inception3a/3 × 3_reduce achieves robust and satisfying performance while maintaining less computational complexity than other combinations. Hence, we concatenate Inception3a/3 × 3 and Inception3a/3 × 3_reduce together as the holistic descriptor.

It matters that computing substantial distances between 175,616-dimensional descriptors is an expensive operation and is a bottleneck of the ConvNet-based place recognition. To reduce the size of the ConvNet descriptors without losing a great accuracy, the redundant and irrelevant features should be omitted to compress the size of descriptors. We perform a random selection [47] of features (i.e., randomly choosing a specific set of elements among the descriptors), which only sacrifices little precision to reduce most of the descriptor size. Our compression proposal is supported by the satisfactory results displayed in Table 2. Finally, we choose the descriptor with the size of 8192 dimensions as the optimal descriptor.

### 4.6. Parameter Tuning of Global Optimization

To achieve the best global optimization performance, the parameters in network flow model need to be tuned to adequate values. The parameters to be tuned are summarized in Table 3. Quantity of flow (*f*) denotes the number of matching images retrieved from database images for a query image. Number of children nodes in graph (k+1) denotes the number of possible database images for the next query image. Cost of edges pointing to hidden nodes (*c*) denotes the threshold of descriptor distance that are used to refuse the corresponding mismatched images.

**Figure 17:**
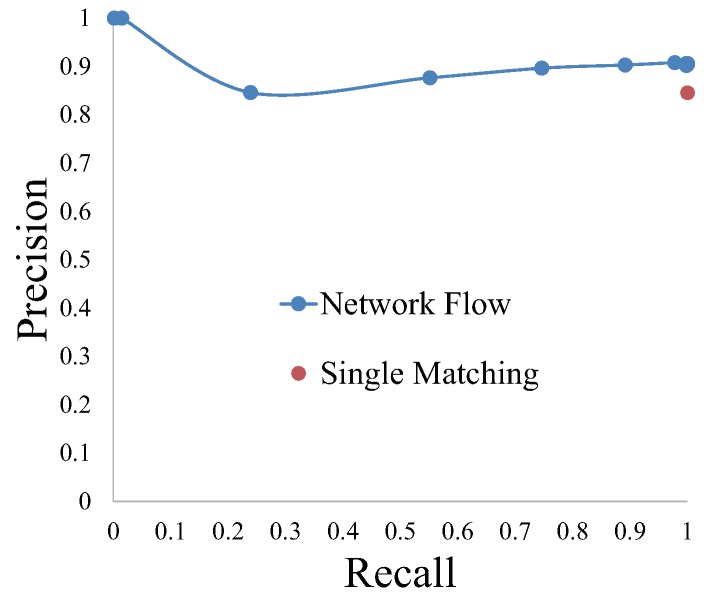
The precision–recall curve derived by tuning parameter *c*, and the precision and recall obtained by single-image matching.

Out of the three parameters, we first determine *f* is 1, which means more than one image could be retrieved from database for a query image. The other two parameters (k+1 and *c*) are determined by the grid search [48] on Bonn dataset. Different values of the two parameters constitute a grid, where the parameter combination that achieves highest precision (defined as Equation (1)) is the optimal parameters. According to the testing on Bonn dataset, the parameter k+1 does not make a difference in a large range, hence k+1 is set to 4. The precisions and recalls using different values of parameter *c* are shown in Figure 17, from which we conclude that c=1.8 is the optimal value. If the parameter *c* is too small, the fluid flow is prone to going through the hidden nodes, so that the recall of image matching is too low. Contrarily, the hidden nodes with too large *c* result in their invalidity. It is obvious that localization by the net flow model is superior to single-image matching.

**Figure 18:**
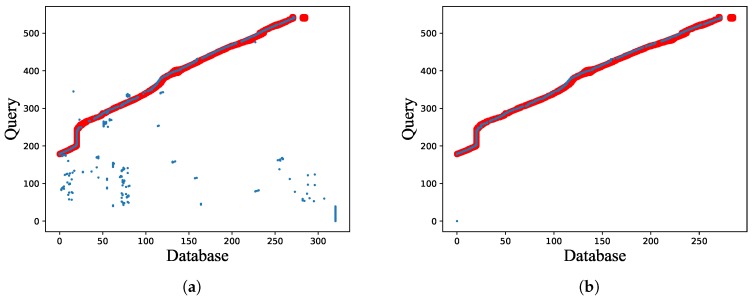
Performance comparison between: (**a**) single image matching; and (**b**) global optimization with network flow on the modified Bonn dataset. The red trajectory denotes the ground truth, and the blue points denote visual localization results.

To validate the superiority of network flow in coping with complicated scenarios, we test the performance of network flow model on a modified dataset, where one-third of images from the start of database are removed. It is to simulate a sporadically occurring situation that the image to be queried does not correspond to any database image. As presented in Figure 18, single image matching does not refuse bad matching results, meanwhile network flow model achieves higher localization precision.

### 4.7. Real-World Experiments

**Figure 19:**
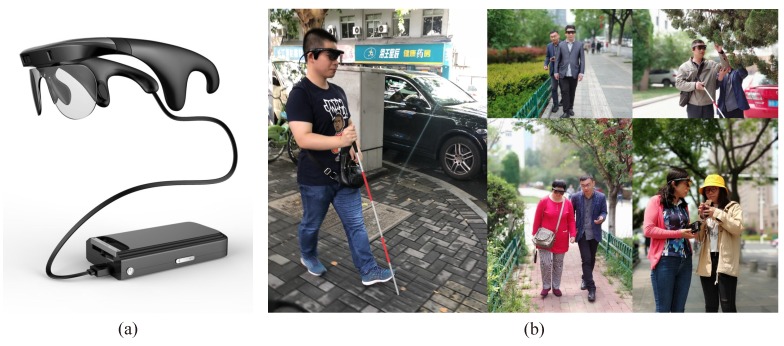
(**a**) Intoer: the wearable assistive devices for visually impaired people; and (**b**) visually impaired volunteers are wearing Intoer to capture images.

To achieve navigational assistance for people with visual impairments, we developed a wearable assistance system Intoer [49], which is comprised of the multi-modal camera RealSense [50], a customized portable processor with GNSS module, and a pair of bone-conduction earphones [51], as shown in Figure 19a. Based on the system, we have previously achieved various assisted utilities, including traversable area and hazard awareness [52,53,54], crosswalks, traffic lights detection [55,56], etc. In this paper, we use the Intoer to capture color images, and the image matching is processed off-line.

**Figure 20:**
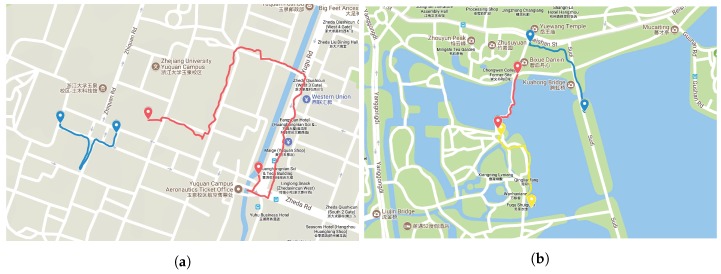
The trajectory of experiments carried out by visually impaired volunteers in: (**a**) the Yuquan Campus of Zhejiang University; and (**b**) the landscape area of the West Lake.

Utilizing the off-the-shelf system, we perform the visual localization experiments in the real-world environments. Five visually impaired volunteers (as shown in Figure 19b) are invited separately to wear Intoer, which is set to autonomously capture color images when the user traverses a route. The routes lie in the Yuquan Campus of Zhejiang University (as shown in Figure 20a) and the landscape area of the West Lake (as shown in Figure 20b), Hangzhou City, China. On those practical routes, the volunteers walk on the sidewalk and encounter pedestrians or vehicles going along or in the opposite direction. Besides, illumination changes, season changes and viewpoint changes exist in the real-world images, which offer the possibility to validate the proposed Visual Localizer in practical environments.

**Figure 21:**
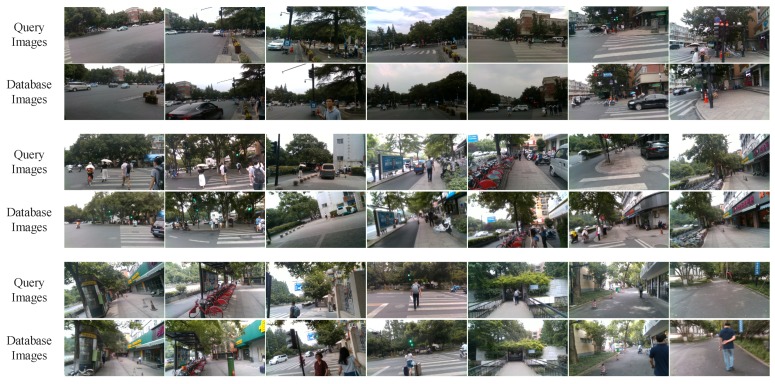
The localization results of a 1000-m trajectory, which is the red route in Figure 20a. Visual localization is achieved under the circumstances of viewpoint changes.

The Visual Localizer’s robustness against viewpoint changes is validated firstly. For the sake of it, both the query images and the database images are captured on the same route by a visually impaired volunteer in a summer afternoon. The localization results are shown in Figure 21. Because the volunteer with visual impairments has no concept of viewpoint, query and database images exhibit viewpoint variations, which are also caused by the movement during visually impaired volunteer’s walking. From the visual localization results, Visual Localizer is robust against viewpoint changes. It further confirms the consumption that the concatenation of Inception3a/3 × 3 and Inception3a/3 × 3_reduce is also viewpoint invariant. With the partial occlusions caused by dynamic objects (e.g., vehicles, pedestrians, etc.), Visual Localizer successfully matches query images with correct database images, as shown in Figure 21.

**Figure 22:**
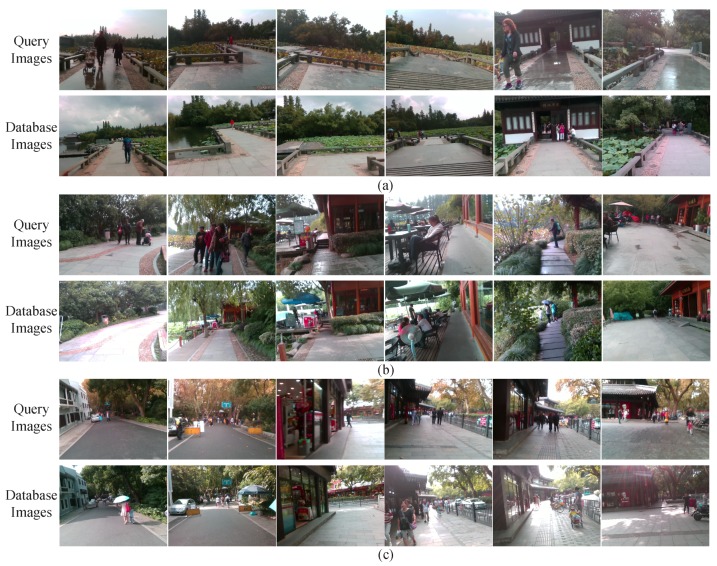
The localization results of experiments carried out by three visually impaired volunteers in the landscape area of the West Lake: (**a**) the visual localization results of the red route in Figure 20b; (**b**) the visual localization results of the yellow route in Figure 20b; and (**c**) the visual localization results of the blue route in Figure 20b.

To validate the robustness against illumination changes and season changes, we invited three volunteers with visual impairments to travel three different routes in the landscape area of the West Lake. The database images were captured on a sunny summer day, while the query images are captured on a rainy winter day. The corresponding visual localization results of the three different routes are presented in Figure 22, from which it is not hard to find the significant examples of image matching under illumination changes. Thereby, it demonstrates that Visual Localizer is robust enough under contrast sunlight intensity. The foliage color changes and vegetation coverage changes appearing in Figure 22a demonstrate that our approach also addresses the visual localization in the cross-season conditions. Furthermore, the appearance changes of the outdoor cafe (Figure 22b) also illustrate the appearance robustness of Visual Localizer. In all of those situations, the visual localization delivers accurate results, even when there are some partial occlusions caused by dynamic objects in the images.

We also performed an experiment to validate the robustness against route changes. The experiment results are presented as Supplementary Material Video S1, where the user firstly visit a recorded route, then the user turn into a new route. When the visually impaired navigator travels into a new route which is not recorded in the database, the query images are no longer matched with any database image. Only if the user returns back to the recorded route, the query images are matched with the corresponding database images again. The localization results under the condition of route changes illustrate that our approach performs well when the user enters a new place.

In general, our approach is robust against viewpoint changes, illumination changes (dim light vs. bright daylight), cross-season changes (winter vs. summer) and route changes. Therefore, Visual Localizer performs well under the visual changes of outdoor assisted navigation.

**Figure 23:**
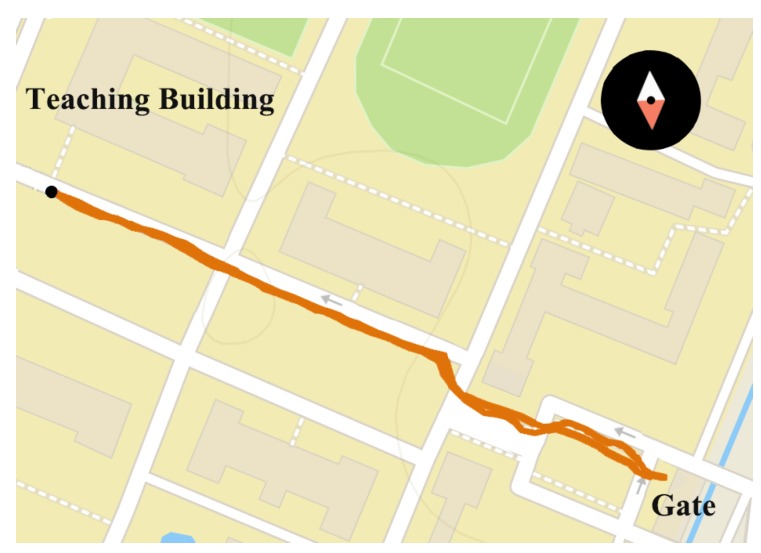
The trajectory of experiments carried out by a volunteer, traveling from a teaching building to the gate of the Yuquan Campus of Zhejiang University. When the camera captured color images, the GNSS module also recorded the longitude and latitude coordinates.

**Figure 24:**
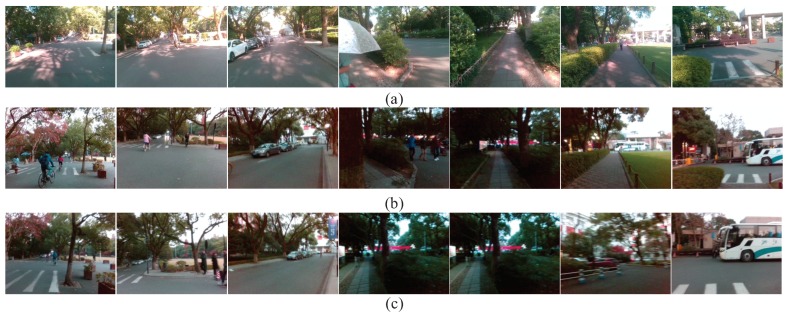
The comparisons of localization results between Visual Localizer and GNSS-based approach: (**a**) query images; (**b**) visual localization results; and (**c**) GNSS-based localization results. The localization results denote the matching images along the orange route as shown in Figure 23.

We performed localization experiments to compare the positioning accuracy of our approach with GNSS-based localization. The route of comparison experiment is shown in Figure 23. The query images were captured on an afternoon in summer, and the database images were captured on an early evening in winter. The query sequence are composed of 255 images, and localization results are shown in Figure 24 and Table 4. Mean error refers to the mean index error between the localization results and the ground truths. Precision is defined as the percentage of matching pairs with index error less than 5. Visual Localizer and GNSS-based localization retrieve 58 and 255 matched positions respectively. Despite Visual Localizer has the lower recall of image matching, the positioning error and precision of Visual Localizer are superior to those of GNSS-based localization.

According to Figure 24, Visual Localizer achieves better localization precision than GNSS-based approach. As shown in the fourth and fifth rows of Figure 24, two query images from different location are matched with the same wrong database image by GNSS-based localization, which is avoided by Visual Localizer.

## 5. Conclusions and Future Work

Aiming to address the problems of viewpoint, appearance and route changes on visual localization, we propose a novel visual localization system—Visual Localizer.

To achieve robust image representation, different layers derived from five prevailing ConvNets are evaluated on their robustness against various environmental changes. We find that the robustness of ConvNet-based descriptor is not positively correlated to the object classification accuracy as well as the filter size. GoogLeNet has overwhelming advantages against other ConvNets because of best performance on both appearance invariance and viewpoint invariance as well as modest computational complexity. Under severe appearance changes, GoogLeNet achieves a precision of more than 60% and a recall of 100%. Data association graph is utilized to organize database and query images, and minimum-cost flow problem is addressed to resolve the best localization results. Applying global optimization of image matching to Visual Localizer promotes the localization precision under route changes.

Our preliminary research has validated the reliability of our proposed assistance system—Intoer—in the application of assisted navigation. Thereby, we utilized the practical images captured by visually impaired volunteers wearing Intoer to validate the performance of Visual Localizer. The results of experiments illustrate that the proposed Visual Localizer performs well in the application of assisted navigation. Currently, image matching is executed off-line. In the future, we plan to achieve effective on-line image matching and to design audio feedback for Visual Localizer on Intoer.

Visual place recognition over perceptually-changing environments generally falls into two categories: utilizing feature representations that are robust to perceptual changes, and learning and predicting appearance changes. In this paper, we have achieved the former task, but there is more work to do beyond Visual Localizer. Future work involves understanding visual information, autonomously labeling key places, and recognizing multiple images captured at the same place as one place. 

## Figures and Tables

**Table 1:** The performance of state-of-the-art ConvNets trained on ImageNet [41] datasets and the assessment on three aspects. The bold and italic items denote the optimums.

Model	ImageNet	Million	Million	Viewpoint	Illumination	Cross-Season
	Accuracy	Multi-Adds	Parameters	Invariant	Invariant	Invariant
AlexNet	57.2%	720	60	Good	Good	Good
VGG16	***71.5%***	15,300	138	Normal	Normal	Bad
***GoogLeNet***	69.8%	1550	6.8	***Best***	***Best***	***Best***
SqueezeNet	57.5%	1700	1.25	Normal	Good	Normal
MobileNet	70.6%	***569***	***4.2***	Normal	Bad	Bad

**Table 2:** Study about the performance of compressed features. The bold and italic items denote the optimums.

Size in Bytes	F1-Score	Percentage of Compression	Percentage of Speedup for Calculating Cosine Distance
175,616	0.8406	0%	0%
131,072	0.8304	25.36%	25.75%
65,536	0.8372	62.68%	36.36%
32,768	0.8338	81.34%	80.31%
16,384	0.8095	90.67%	89.39%
***8192***	***0.8131***	***95.34%***	***93.94%***
4096	0.7889	97.67%	96.97%

**Table 3:** Parameters tuning in the minimum-cost flow model.

Parameter	Denotation
*f*	quantity of flow
k+1	number of children nodes
*c*	cost of edges pointing to hidden nodes

**Table 4:** The performance comparison between Visual Localizer and GNSS-based localization. The bold and italic items denote the optimums.

	Visual Localizer	GNSS-Based
Mean Error	***7.95***	24.09
Precision	***89.66%***	39.22%
Matched Number	58	***255***

## Supplementary Materials

The following is available online at http://www.mdpi.com/1424-8220/18/8/2476/s1, Video S1: Performance of Visual Localizer on route changes.

## Abbreviations

The following abbreviations are used in this manuscript:

ABLE	Able for Binary-appearance Loop-closure Evaluation
BoW	Bag of Words
ConvNet	Convolutional Neural Network
FOV	Field Of View
GIS	Geographic Information System
GNSS	Global Navigation Satellite System
HOG	Histogram of Oriented Gradient
LDB	Local Difference Binary
MRoI	Multiple Regions of Interest
QR	Quick Response
RFID	Radio Frequency IDentification
SLAM	Simultaneous Localization And Mapping
SURF	Speeded Up Robust Features
